# *Elaeis oleifera* Genomic-SSR Markers: Exploitation in Oil Palm Germplasm Diversity and Cross-Amplification in Arecaceae

**DOI:** 10.3390/ijms13044069

**Published:** 2012-03-28

**Authors:** Noorhariza Mohd Zaki, Rajinder Singh, Rozana Rosli, Ismanizan Ismail

**Affiliations:** 1 Advanced Biotechnology and Breeding Centre, Malaysian Palm Oil Board (MPOB), P.O. Box 10620, Kuala Lumpur 50720, Malaysia; E-Mails: rajinder@mpob.gov.my (R.S.); lizana@mpob.gov.my (R.R.); 2 School of Bioscience and Biotechnology, Faculty Science and Technology, Universiti Kebangsaan Malaysia, Bangi 43600, Selangor, Malaysia; E-Mail: maniz@ukm.my

**Keywords:** simple sequence repeat (SSR), *Elaeis oleifera*, genomic library, transferability

## Abstract

Species-specific simple sequence repeat (SSR) markers are favored for genetic studies and marker-assisted selection (MAS) breeding for oil palm genetic improvement. This report characterizes 20 SSR markers from an *Elaeis oleifera* genomic library (gSSR). Characterization of the repeat type in 2000 sequences revealed a high percentage of di-nucleotides (63.6%), followed by tri-nucleotides (24.2%). Primer pairs were successfully designed for 394 of the *E. oleifera* gSSRs. Subsequent analysis showed the ability of the 20 selected *E. oleifera* gSSR markers to reveal genetic diversity in the genus *Elaeis*. The average Polymorphism Information Content (PIC) value for the SSRs was 0.402, with the tri-repeats showing the highest average PIC (0.626). Low values of observed heterozygosity (*H_o_*) (0.164) and highly positive fixation indices (*F_is_*) in the *E. oleifera* germplasm collection, compared to the *E. guineensis*, indicated an excess of homozygosity in *E. oleifera*. The transferability of the markers to closely related palms, *Elaeis guineensis*, *Cocos nucifera* and ornamental palms is also reported. Sequencing the amplicons of three selected *E. oleifera* gSSRs across both species and palm taxa revealed variations in the repeat-units. The study showed the potential of *E. oleifera* gSSR markers to reveal genetic diversity in the genus *Elaeis*. The markers are also a valuable genetic resource for studying *E. oleifera* and other genus in the Arecaceae family.

## 1. Introduction

*Elaeis oleifera* is a species in the oil palm genus along with the commercial *Elaeis guineensis* and occurs naturally in South-Central America, from Honduras to Colombia and in the Amazon region [1]. This American species is seen as a promising genetic resource for oil palm improvement and is currently used in oil palm hybrid (*E. guineensis* × *E. oleifera*) breeding programs. It has attracted the attention of breeders by reason of several interesting agronomic traits: low height increment, resistance to *Fusarium* wilt and lethal yellowing [2], which can have important economic implications if introgressed into *E. guineensis*. Beside the agronomic traits, the oil from *E. oleifera* is highly unsaturated (*i.e.*, high iodine value, or IV) with high linoleic and oleic acids, low palmitic acid and high carotene [3]. A genomic *in situ* hybridization technique (GISH) using specific DNA probes to distinguish *oleifera* and *guineensis* chromosomes has been developed to assist hybrid backcross breeding programs [4].

In plant genetics and breeding studies, DNA-based assays, and especially molecular markers, are known to be efficient tools for genetic diversity assessment, molecular ecology studies, gene mapping as well as marker-assisted selection (MAS) [5]. Among all the available molecular markers, simple sequence repeats (SSR) are still among the most favored, due to their many desirable attributes, which include hypervariability, wide genomic distribution, co-dominant inheritance, a multi-allelic nature and chromosome specific location. In addition, they are easily assayed using PCR [6]. Currently, SSRs also appear to be the most promising molecular marker systems for understanding oil palm population genetic structure [7]. Furthermore, SSR markers which are highly transferable across taxa are advantageous as they save time and cost in developing SSR markers for members of taxa that have not been extensively studied. These SSR markers are also useful tools for comparative genetic studies within the genus. In oil palm, *E. guineensis*-based SSR markers have been used to construct genetic maps [8,9], and are also actively used to characterize germplasm collections [1].

The Malaysian Palm Oil Board (MPOB) has an extensive collection of germplasm from both species of oil palm. *E. guineensis* from Africa and *E. oleifera* maintained as *ex-situ* collections in Kluang, Johor, Malaysia. Assessing the performance and genetic diversity of the wild material is important for understanding the genetic structure of natural oil palm populations. Furthermore, the information is important for oil palm breeding programs, and also for continued *ex-situ* conservation of the germplasm in Malaysia. Currently, only the *E. guineensis* germplasm is well characterized, using various types of molecular markers, such as isozymes [10], restriction fragment length polymorphisms (RFLPs) [11], amplified fragment length polymorphism (AFLP) [12], random amplified polymorphic DNA (RAPD) [13] and SSRs [7,14]. However, the work on *E. oleifera* has been limited, only involving RAPD [15] and SSR markers developed from *E. guineensis* [14,16]. Nevertheless, the increasing number of sequence collections available for *E. oleifera* has made it possible to develop SSR markers from *E. oleifera* and utilize them to understand the genetics of the species.

Thus, the objectives of this study were to (a) develop and characterize *E. oleifera* genomic SSR markers from a collection of *E. oleifera* genomic sequences; (b) evaluate the efficiency of these markers in assessing the genetic diversity in the MPOB *E. oleifera* germplasm collection; and (c) determine the transferability of *E. oleifera* SSR markers among selected palm genera and taxa.

## 2. Results and Discussion

### 2.1. Characterization of *E. oleifera* Genomic SSRs

The *GeneThresher*™ library is a comprehensive collection of gene sequences of oil palm obtained from sequencing of the hypomethylated region of the oil palm genome using methylation filtration technology [17]. As such, the sequences are likely to be located within or close to the genic regions in oil palm. The clear advantage is that the SSR locus may point to a gene of interest and show high levels of polymorphism associated with being genomic-based SSR markers. Of the 2000 *E. oleifera GeneThresher*™ derived sequences used in this study, 1861 non-redundant sequences (1735 singleton and 126 consensus) were successfully assembled with CAP3 sequence assembly software [18]. A total of 603 SSRs were identified in 472 genomic sequences, suggesting the *E. oleifera* genomic library is a valuable resource for this genetic marker type.

One hundred and four (22%) of the genomic sequences contained more than one SSR. Mononucleotides were the most abundant repeat type (437 = 72.4%), and showed a strong bias to the A/T repeat-motifs (97.9%) over the C/G repeat motif (Table 1). Feng *et al*. [5] reported that mononucleotides were generally not very informative and thus were not considered for analysis in this study. With the omission of mononucleotides, the most prevalent repeats were di-nucleotides (63.3%), followed by tri-nucleotides (24.2%), tetra-nucleotides (6%), penta-nucleotides (4.8%), hexa-nucleotides (0.6%) and 1.2% of the hepta-nucleotides. Among the di-nucleotide repeats, the AG/CT (46.7%) and AT/AT motifs (43.8%) were by far the most common, while AC/GT was present in low abundance (9.5%). The abundance of the AG/CT motif has consistently been reported in EST sequences from *E. guineensis* [7,19,14], peach [20], coffee [21] and rubber [5]. AG/CT SSR may have a higher probability of being linked to important traits [22], based on report by Morgante *et al*. [23], highlighting the frequent occurrence of this di-repeat motif in the 5′ flanking regions of genes in plants. The two most common tri-nucleotide motifs were AAG/CTT (50%) and AAT/ATT (25%) followed by AGG/CCT (17.5%), AAC/GTT (5%) and ACC/GGT (2.5%). The abundance of the AAG/CTT tri-repeat motif in *E. oleifera* is similar to that reported for *E. guineensis* EST-SRR [7,14,19], *Arabidopsis thaliana* [24], soybean [25], barley [26] as well as coffee [27]. The most abundant tetra- and penta-nucleotide repeat-motifs were AAAT/ATTT and AAAAG/CTTTT at a frequency of 40% and 50%, respectively.

### 2.2. Primers Designed for *E. oleifera* gSSR

With exclusion of the 437 mononucleotide repeats, attempts were made to design primer pairs for the 166 identified SSRs. Primer pairs were successfully designed for 144 SSRs (86.7%), of which 63.9% were di-repeats, 24.3% tri-repeats, 4.9% tetra and penta-repeats each, 0.7% hexa-repeats and 1.4% hepta-repeats. The failure to design primers for the remaining sequences (13.3%) was probably due to short (or absence of) flanking regions, or that the sequences submitted did not correspond to the minimum criteria required by the primer design software [7]. Nevertheless, the success rate is high compared to previous work on genomic SSRs of wheat [28] and *Sorghum* [29], where the success rates were only 51% to 66%. Subsequently, 20 of the 144 *E. oleifera*-based gSSR primer pairs (Table 2), representing a variety of motifs (di- to penta-repeats) were randomly selected to analyze samples from the oil palm germplasm collection.

### 2.3. Germplasm Characterization: Allelic Polymorphism and Genetic Variation in *E. oleifera* and *E. guineensis*

To ascertain the attributes of the *E. oleifera*-based gSSR markers in characterizing *E. oleifera* germplasm, 20 primer pairs (markers) were tested on a panel of 119 *E. oleifera* palms from the germplasm collection. Ten *E. guineensis* from the Nigerian collection and another 10 from the MPOB advanced breeding material population (Deli *dura*) were included for comparison. This allowed the study to also determine the ability of the *E. oleifera*-derived SSR markers to reveal the genetic diversity in the Deli *dura* material which had undergone several cycles of self-pollination, and also the wild Nigerian materials. This provenance is reported to be the center of diversity for *E. guineensis* [11].

Eighteen of the 20 primers successfully produced amplicons (Table 2), and 15 of the 18 primers (83.3%) reveal polymorphisms in at least one of the collections analyzed. The remaining three, sMo00108, sMo00140 and sMo00161 were monomorphic in all the samples tested. The high level of detected polymorphism (83.3%) shows the ability of *E. oleifera* gSSR markers to amplify the target sequences and detect polymorphism in both *Elaeis* palms.

The *E. oleifera* gSSRs detected 89 alleles, ranging from 1 to 13 across the *Elaeis* samples. Of them (alleles), 48.3% and 31.5% of alleles were specific to *E. oleifera* and *E. guineensis,* respectively, and 20.2% common in both species. Within the repeats, tri-nucleotides detected more alleles (mean = 7.8 alleles) than the other repeats. It would appear that the tri-nucleotide genomic SSRs show higher average PIC values than di-nucleotide repeats. This is most likely a reflection of the specific region of the genome targeted by the methylation filtration technique. Botstein *et al*. [30] defined any locus (marker) with PIC > 0.5 as highly polymorphic. All the loci derived from the di- and tri-repeat gSSRs met this criterion, except sMo00055 and sMo00132. This shows that both the repeat types are generally informative in the samples analyzed. However, the mean PIC (0.402) from this study was slightly lower than that previously reported for *E. guineensis*-derived EST-SSRs (7, mean = 0.53; and 14, mean = 0.65) which were used mainly to analyze *E. guineensis* germplasm.

Interestingly, the ability of *E. oleifera*-derived gSSRs to reveal allelic polymorphism and genetic diversity in the *Elaeis* genus was more efficient than by other tested marker systems. For instance, *E. oleifera* gSSRs generated more alleles (*A_o_*) in both *Elaeis* species (means = 2.27–2.66; Table 3), compared to RFLP [11] and isozyme [10,31], which generated *A_o_* < 2.0. Furthermore, the efficiency of the *oleifera* genomic SSRs in revealing heterozygosity was distinctively higher (mean *H_e_* = 0.273) than in previous studies on *E. guineensis* using isozymes (*H_e_* = 0.184, 10), RFLP (*H_e_* = 0.135, 15; *H*_e_ = 0.199, 11) and AFLP (*H*_e_ = 0.117, 15).

By focusing only on analysis carried out on *E. oleifera* collections, *H_e_* revealed by *E. oleifera* SSR markers (0.262) was slightly lower than those generated by *E. guineensis* EST-SSR markers (14; *H_e_* = 0.286). As such, *E. oleifera* gSSR markers are also additional promising tools for characterizing *E. oleifera* collections, although the *A_o_* and *H_e_* values are lower than those generated by *E. guineensis* gSSRs (16; *A_o_* = 0.535 and *H_e_* = 0.69). The differences revealed by both the genomic SSR markers were possibly due to the number of samples analyzed and populations evaluated. Billotte *et al*. [16] analyzed 21 *E. oleifera* samples (1–2 samples per country), whereas 119 *E. oleifera* samples (22–34 samples per country) were analyzed in this study. Furthermore, the genomic library utilized in this study was constructed from hypo-methylated regions, the chances of the employed *E. oleifera* gSSR markers being closely located within the conserved coding regions are higher than in the sequences obtained from a conventional genomic library. This could also explain the lower diversity observed in this study.

Regarding the genetic variation between the two *Elaeis* species, the heterozygosity in the *E. oleifera* germplasm varied from 0.102 to 0.200 (mean *H_o_* = 0.164), while *E. guineensis* generated *H_o_* from 0.118 (Deli *dura*) to 0.321 (Nigeria) (mean *H_o_* = 0.220). *E. oleifera* generally had lower diversity, compared to *E. guineensis*, with higher *H_o_* revealed by the Colombian and Panama palms (*H_o_* = 0.200; *H_o_* *=* 0.193 respectively). The higher *H_o_* in both collections could be due to human assisted movement of palm samples that was probably accelerated during and subsequent to construction of the Panama Canal in 1914. Some palms could have been brought in from other South American countries, widening the genetic base of *E. oleifera* in these countries. Furthermore, the *H_e_* value obtained for *E. oleifera* (mean = 0.262) was comparable to those obtained by RFLP (*H_e_* = 0.225) and AFLP (*H_e_* = 0.298) analyses in screening 241 *E. oleifera* accessions [15]. Among *E. guineensis*, *H_e_* for the Nigerian samples (0.329) was lower than that obtained with *E. guineensis* EST-SSR markers [7,14], where the reported *H_e_* values were 0.442 and 0.534 respectively.

The *F_is_* values were positive at all loci in all tested collections with mean *F_is_* ranging from 0.024 (Nigeria) to 0.546 (Deli *dura*) (Table 3). This reflects the differences in the prospecting areas for the germplasm. The *E. guineensis* germplasm was collected over widespread areas in Africa, resulting in more heterogeneous collections compared to *E. oleifera,* which were mostly from scattered isolated populations across four South-Central American countries [32]. This may have encouraged inbreeding, resulting in a relatively homozygous genome for the *E. oleifera* collections. Furthermore, the extremely high *F_is_* in Deli *dura* populations compared to the Nigerian and other *E. oleifera* germplasm supported the low level of genetic diversity of this advanced breeding population which had undergone several cycles of selfing. This also explains the low genetic diversity of Deli *dura* population (mean *H_e_* = 0.260) generated by *E. oleifera* gSSR markers in this study, which was even lower than that revealed by *E. guineensis* EST-SSR markers (14; mean *H_e_* = 0.340).

### 2.4. Genetic Relationship of the Genus *Elaeis*

**Figure 1:**
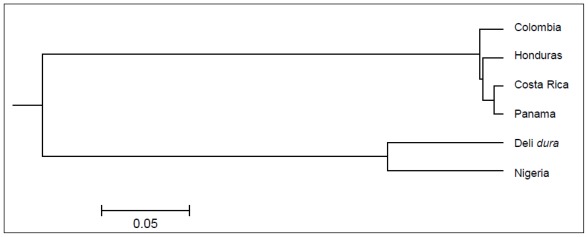
Unweighted pair group with arithmetic mean (UPGMA) dendrogram reflecting genetic relationship among the *Elaeis* genus revealed by 18 *Elaeis oleifera* gSSR markers.

The 18 informative *E. oleifera* genomic SSRs described in this study successfully grouped the six collections of oil palm into two distinct clusters: *E. oleifera* and *E. guineensis* (Figure 1). In general, the clusters supported the origins and geographical distributions of the palms, *E. oleifera* from Latin America and *E. guineensis* from Africa. Within *E. oleifera*, the collections from Costa Rica and Panama showed a very close relationship. This is not surprising as Costa Rica and Panama are neighboring countries. The collection from Honduras also fell into the same cluster as Costa Rica and Panama, again probably due to the close proximity of Honduras to Costa Rica and Panama. The collections from Colombia were clearly separate from the other three collections from Central America.

### 2.5. Cross-Transferability of *E. oleifera* gSSR Markers

Eleven of the *E. oleifera* gSSR markers produced clear and prominent banding profiles in both *E. guineensis* and *E. oleifera*. These markers were further used to evaluate cross species/genera transferability in the Arecaceae taxa (Table 4). Successful amplification (transferability) of either similar or varying sized fragments was obtained with all the primers in the coconut palms and in at least one of the tested ornamental palms species. As such, the *E. oleifera* gSSR markers showed 100% transferability to *E. guineensis* and, more importantly, also perfect (100%) transferability in the tested *Cocos nucifera* samples. With the ornamental palms, the frequencies of transferability were *Euterpe* (72.7%) *> Oenocarpus* (63.6%) *> Jessinia* (54.5%) *> Ptychosperma* (54.5%) *> Dictyosperma* (45.5%) *> Cyrtostachys* (45.5%). Two markers: sMo00055 (di-repeats) and sMo00137 (penta-repeats), generated clear banding profiles of various sizes in all the samples analyzed, including the ornamental palms.

SSR primers developed for one species are known to often detect homologous sites in related species. The ability of sMo00055 and sMo00137 markers to amplify fragments with similar sizes indicates their efficiency in revealing sequence conservation among the species in the Arecaceae family. In general, cross species transferability differs highly among taxa, especially in flowering plants [33]. The transferability across related species and genus facilitates comparative genetic studies [34]. The successful rate of transfer for SSR has been reported to average 76.4% at the genus level and 35.2% at the family level [35]. The success rate for *E. oleifera* SSRs averaged 75% at the genus level, comparable to *Phyllostachys Pubescens* (75.3%), but lower than rice (90%) [36]. Furthermore, all the tested *Elaeis*-derived gSSR markers were able to amplify PCR products in *Cocos*, reflecting their capability in characterizing the three different *Cocos* samples tested. This also suggests the relatively close proximity of *E. oleifera* to coconut. The high cross-transferability of *E. oleifera* gSSR markers to *Elaeis* species and related genera suggests the potential application of these markers in comparative studies across members of the Arecaceae family. High cross-species conservation of SSR loci within genus has also been reported for *Olea* [37], *Picea* [38] and *Pinus* [39].

### 2.6. Sequence Variability and Molecular Basis of *E. oleifera* gSSR Markers Fragment Length Polymorphism

**Figure 2:**
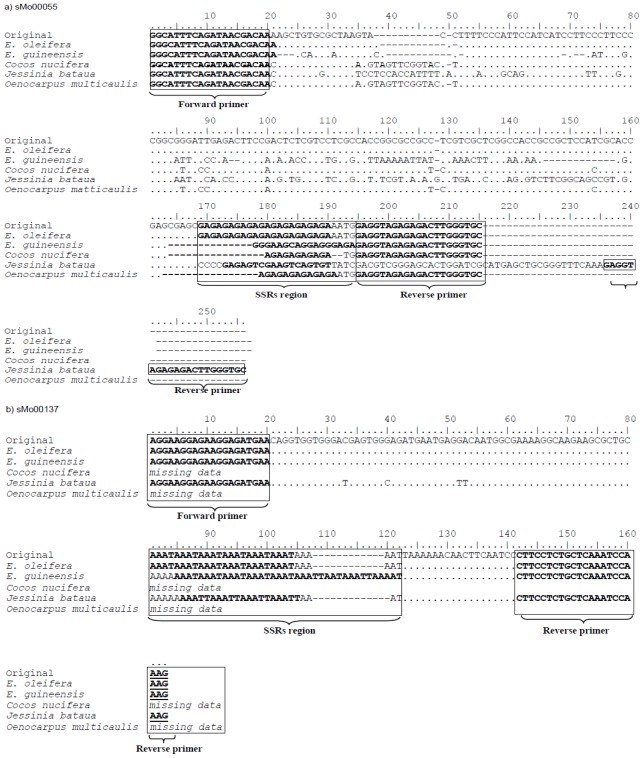

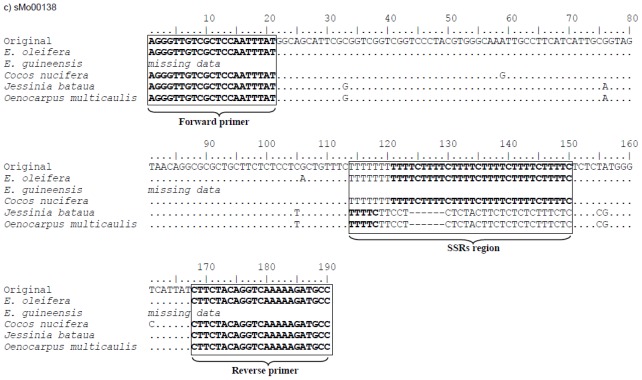
ClustalW alignments of sequences obtained from PCR bands amplified by the *E. oleifera* gSSR markers (**a**) sMo00055; (**b**) sMo00137; and (**c**) sMo00138 in *E. oleifera*, *E. guineensis*, *C. nucifera*, *Jessinia bataua* and *Oenocarpus multicaulis*. Missing data refers to amplicons that were not successfully cloned.

Three markers comprising various repeat types (sMo00055/di-repeat, sMo00137/tetra-repeat and sMo00138/penta-repeat) were used to determine the sequence variability in some of the species in Arecaceae family (*E. oleifera*, *E. guinensis*, *Cocos nucifera*, *Jessinia bataua* and *Oenocarpus multicaulis*). Amplified PCR fragments of these markers in selected individuals were cloned and sequenced. The amplicons of the three SSR markers were successfully cloned and sequenced. The sequences were aligned with the original sequence from which the primers were designed (Figure 2). In general, sMo00137 gave the highest sequence similarity among the samples analyzed, followed by sMo00138. sMo00055 showed the lowest similarity with highest number of bases interrupted in the flanking region.

Generally, the sequence data generated by the three selected *E. oleifera* gSSR loci (sMo00055, sMo00137 and sMo00138) revealed variable numbers of repeat motifs in the SSR regions within the tested samples. This further explains the primary basis of the observed fragment length polymorphism in the Arecaceae family screened in this study. The variations were mainly due to changes in the number of repeat motifs in the SSR region, combined with indels and base substitutions. Similar results were reported by Billotte *et al*. [16] and Ting *et al*. [14] who employed *E. guineensis* SSR and EST-SSR markers, respectively. However, looking specifically at sMo000555, the repeat motif observed in *E. oleifera* was missing in *E. guineensis*, and the repeat number was very low in coconut and one of the ornamental palms. Although this locus was successfully amplified in all samples, the lack of repeat conservation in some samples suggests that the amplified fragments may not represent functional SSRs in those species. Nevertheless, the ability of *E. oleifera* genomic SSRs (sMo00137 and sMo00138) to reveal high inter-species and inter-genera transferability (>90%) supports the close phylogenetic relationship between the species and genera.

## 3. Experimental Section

### 3.1. Plant Materials and gSSR Source

The oil palm germplasm collections used in this study are maintained at the MPOB Research Station, Kluang, Johor. A total 149 spear leaves (one per palm) were harvested from the palms in Table 5. Cross-transferability of the *E. oleifera* gSSR was tested on three coconut (*Cocos nucifera*) samples and six ornamental palms (*Euterpe oleracea*, *Jessinia bataua*, *Oenocarpus multicaulis*, *Ptychosperma macarthurii*, *Cyrtostachys renda* and *Dictyosperma album*). Genomic DNA was extracted and purified from each spear leaf using the modified CTAB method described by Doyle and Doyle [40]. The *E. oleifera* genomic library was constructed using *GeneThresher*™ Technology (17) and the genomic clones and sequences stored at MPOB’s Biological Resource Centre (MBRC).

### 3.2. SSR Identification and Primer Design

A total of 2000 *E. oleifera* genomic sequences were assembled using the CAP3 assembly program [18] with default parameters. The file containing the sequences was submitted in a FASTA formatted text file. Identification and localization of the SSR markers were performed using MISA software as described by Thiel *et al.* [26]. The search criteria were: mononucleotides ≥10 repeat units, di-nucleotides ≥7 repeat units and tri-, tetra-, penta- and hexa-nucleotides ≥5 repeat units respectively. Interrupted compound SSRs were also selected where the interval bases interrupting two SSRs were ≤10 repeat units. The relative frequency and distribution of the repeat types in the genomic sequences were estimated. Primer pairs were designed flanking the identified SSRs using PRIMER 3 [41]; all the primers were synthesized by Invitrogen ™ USA.

### 3.3. SSR Analysis

The forward primer was 5′ end-labeled in 1 μL reaction containing 4.5 μM forward primer, 0.1 μL γ-^33^p dATP (GE Healthcare Biosciences, UK, 3000Ci/mmol) and 1U T4 polynucleotide kinase (Invitrogen™ USA) for 1 hour at 37 °C. The PCR reaction was subsequently carried out in 10 μL of 1 μL 10X PCR buffer (buffer composition-MgCl_2_), 15 mM MgCl_2_, 1 mM dNTPs, 5 μM unlabeled reverse primer, 1 μL labeled forward primer, 0.5 U *Taq* DNA polymerase and 50 ng template DNA. PCR was performed in a Perkin Elmer 9600 thermocycler as follows: denaturation at 95 °C for 3 min, 35 cycles at 95 °C for 30 s, 52–56 °C for 30 s (depending on the primers requirement), 72 °C for 30 s and a final extension at 72 °C for 5 min. The PCR reaction was stopped by addition of 10 μL formamide dye (0.3% bromophenol blue, 0.3% xylene cyanol, 10 mM EDTA pH 8.0, 97.5% deionized formamide). A total 5 μL of the mixture was denatured at 90 °C for 3 min, chilled on ice, and separated in a 6.0% polyacrylamide gel containing 7 M urea in 0.5 X TBE buffer at constant power of 1600V for 3 hours. The gel was then dried and exposed to X-ray film (Kodak) for 3–4 days at −80 °C. The size of each allele was determined using the 100–330 bp AFLP DNA ladder (Invitrogen™ USA).

### 3.4. Data Analysis

Only fragments that could be clearly scored were used in the data analysis. The genotyped data were analyzed using POPGENE version 1.32 [42]. The genetic diversity parameters analyzed for included: percentage of polymorphic loci (0.95 criterion) (*P*), expected and observed heterozygosity (*H_e_* and *H_o_*) in the collections used and *fixation indices* (*F_is_*). Chi squared tests were performed for each locus for deviation of the genotypes from the Hardy-Weinberg equilibrium (*HWE*). The allelic polymorphism information content (PIC) for each gSSR marker and distance matrix [43] between the populations were calculated using the PowerMarker V3.25 software [44]. The unweighted pair-group method with arithmetic averaging (UPGMA) [45] dendrogram was constructed from the distance matrix [43] imported from PowerMarker V3.25 using MEGA4 [46].

### 3.5. Cross-Transferability Amplification

Eleven *E. oleifera* gSSR markers that produced clear banding profiles in both *E. guineensis* and *E. oleifera* samples were further used to study cross species and genus amplification within Arecaceae family. The markers were tested against three *Cocos nucifera* varieties and six ornamental palms (Table 5). The SSR analysis, as described above, was carried out at least twice to confirm the transferability of the primers.

### 3.6. Sequencing of Cloned SSR-PCR Products for Alignment and Phenetic Analysis

The amplicons generated by three selected *E. oleifera* gSSR markers (sMo00055, sMo00137 and sMo00138) in the *E. oleifera*, *E. guineensis*, *Cocos nucifera*, *Jessinia bataua* and *Oenocarpus multicaulis* samples were excised from the agarose gel and purified. The purified fragments were cloned into pCR2.1-TOPO vector (TOPO TA cloning kit, Invitrogen™ USA) and sequenced using the ABI PRISM 377 automated DNA sequencer. The sequences were aligned and compared using CLUSTALW multiple sequence alignment tool employing BIOEDIT sequence alignment editor version 7.0.0 [47]. The sequences were also compared to the original genomic sequence containing the SSR.

## 4. Conclusions

A set of *E. oleifera* gSSR markers developed were found to be valuable genetic resources for understanding the genetic diversity of *E. oleifera* and *E. guineensis*. The study indicates that *E. oleifera-*derived SSR markers were more efficient in revealing the genetic diversity of *E. oleifera* than *E. guineensis* EST-SSR markers. The sequence data showed their ability to amplify DNA, not only in the two oil palm species, but also in coconut and other selected ornamental palms, thus verifying the ability of SSRs to amplify across species and genera in the Arecaceae family. Furthermore, the variability in allele sizes and sequences among the species reflected the mutational processes that had taken place at both the repeat and flanking regions. An expanded study using all the available SSR markers on a larger set of samples (from both species of oil palm) would provide a clearer picture on the genetic diversity of the germplasm available at MPOB

## Figures and Tables

**Table 1:** Frequency and distribution of SSRs in 2000 *Elaeis oleifera* genomic sequences.

SSR Motif	Number of Repeat Units												Total

	5	6	7	8	9	10	11	12	13	14	15	>15	
Mononucleotide													
A/T	-	-	-	-	-	101	78	60	37	29	28	95	428
C/G	-	-	-	-	-	2	3	-	1	-	-	3	9
Di-nucleotide													
AC/GT	-	-	4	2	1	2		1	-	-	-	-	10
AG/CT	-	-	7	8	7	3	9	1	1	6	2	5	49
AT/AT	-	-	6	5	5	6	6	2	2	-	2	12	46
Tri-nucleotide													
AAC/GTT	-	2	-	-	-	-	-	-	-	-	-	-	2
AAG/CTT	9	4	3	-	1		1	1	1	-	-	-	20
AAT/ATT	1	3	2	1	-	1		1	1	-	-	-	10
ACC/GGT	-	1	-	-	-	-	-	-	-	-	-	-	1
AGG/CCT	5	2	-	-	-	-	-	-	-	-	-	-	7
Tetra-nucleotide													
AAAC/GTTT	-	-	-	1	-	-	-	-	-	-	-	-	1
AAAG/CTTT	1	-	-		-	-	-	-	-	-	-	-	1
AAAT/ATTT	1	3	-		-	-	-	-	-	-	-	-	4
AATT/AATT	-	1	-		-	-	-	-	-	-	-	-	1
ACAT/ATGT	-	-	-	1	-	1	-	-	-	-	-	-	2
AGCT/ATCG	1	-	-	-	-	-	-	-	-	-	-	-	1
Penta-nucleotide													
AAAAG/CTTTT	3	1	-	-	-	-	-	-	-	-	-	-	4
AAAAT/ATTTT	3	-	-	-	-	-	-	-	-	-	-	-	3
AGGGG/CCCCT	1	-	-	-	-	-	-	-	-	-	-	-	1
Hexa-nucleotide													
AGAGGG/CCCTCT	1	-	-	-	-	-	-	-	-	-	-	-	1
Hepta-nukleotide													
AAACCCT/ATTTGGG	-	-	-	-	-	-	-	-	-	-	-	2	2
N (Mono-)	-	-	-	-	-	103	81	60	38	29	28	99	437
NN (Di-)	-	-	17	15	13	11	15	4	3	6	4	17	105
NNN (Tri-)	15	12	5	1	1	1	1	2	2	-	-	-	40
NNNN (Tetra-)	3	4	-	2	-	1	-	-	-	-	-	-	10
NNNNN (Penta-)	7	1	-	-	-	-	-	-	-	-	-	-	8
NNNNNN (Heksa-)	1	-	-	-	-	-	-	-	-	-	-	-	1
NNNNNNN(Hepta-)	-	-	-	-	-	-	-	-	-	-	-	2	2
Total													603

**Table 2:** Information on the *E. oleifera* gSSR markers used for germplasm analysis and cross- transferability evaluation.

Primer ID	Primer Sequence (5′-3′) (F: Forward; R: Reverse)	SSR Motif	Ta (°C)	Amplicon (bp)	Accession No. (ProbeDB)	Allele No.	PIC
**Di-nucleotide**							
sMo00018	F: TTAAATGAGAGAGAGACGAGGAC R: TGGAGCCATGAGAAAGAGTA	(CT)_14_	54	246	Pr009947963	6	0.555
sMo00020	F: CCTTTCTCTCCCTCTCCTTTTG R: CCTCCCTCCCTCTCACCATA	(AG)_15_	58	190	Pr009947964	12	0.824
sMo00024	F: TCACCAAAGCAGAAGAAACA R: GGTGTTGATAATTGCCTGAA	(AT)_28_	54	223	Pr010315683	-	-
sMo00027	F: TTACAGTTGAGGCAGTATGTCAAT R: CTGTATGTCAAACCTTCTGCAC	(TC)_14_	50	209	Pr009947965	6	0.574
sMo00055	F: GGCATTTCAGATAACGACAAA R: GCACCCAAGTCTCTCTACCTC	(GA)_11_	54	202	Pr010315684	5	0.243
sMo00108	F: AGCTTCAATTCATACGCAAC R: TGTTATATGTGACTACCAGAGCA	(AT)_19_	53	170	Pr010315685	1	0
Mean						**6.0**	**0.549**
**Tri-nucleotide**							
sMo00127	F: GTGGTTTGGGAGAAAGAGTGT R: TGCGGTGGATTAGCATTATT	(GAA)_12_	56	205	Pr010315686	-	-
sMo00128	F: TAGCTCCAACAGCTTGCCTTAT R: GGTCCCGTCCTATGATTTATTCT	(AAT)_12_	56	192	Pr009947966	6	0.654
sMo00129	F: TTAGTATTGGGTGTGCATAAGTGG R: GCTTCCAGCTCCTCTTTCTACC	(TTC)_13_	56	229	Pr009947967	8	0.786
sMo00130	F: TAAGCAAAAGATCAGGGCACTC R: GGCTGGTGAAAATAGGTTTACAAAG	(AAG)_11_	56	192	Pr009947968	13	0.801
sMo00132	F: ATAGCCAGAGGGCAAAACTGT R: GCAACACACGGACTCAAAACTA	(TTA)_13_	56	161	Pr009947969	4	0.264
Mean						**7.8**	**0.626**
**Tetra-nucleotide**							
sMo00134	F: TCCCAATAGTCGTTACAAACCAG R: GATTAGCAAAAGGGCAAAAAGG	(ATTA)_6_	56	252	Pr009947970	2	0.338
sMo00137	F: AGGAAGGAGAAGGAGATGAACAG R: CTTTGGATTTGAGCAGAGGAAG	(AAAT)_6_	54	151	Pr010315687	3	0.141
Mean						**2.5**	**0.240**
**Penta-nucleotide**							
sMo00138	F: AGGGTTGTCGCTCCAATTTAT R: GGCATCTTTTTGACCTGTAGAAG	(TTTTC)_6_	56	190	Pr009947971	5	0.498
sMo00140	F: TTAGATCATTTCCCTTGCTTCG R: CGCTGGTCCTGATAACACATT	(AAAAT)_5_	56	216	Pr010315688	1	0
sMo00141	F: ACTTGACATACAGGTTCCACTGA R: CCTGCTACCTCCTAATTCTATCAAA	(TTCTT)_5_	56	174	Pr010317029	2	0.218
sMo00147	F: TACCCAATCCCACCGAGTTA R: CGTCTCCACTGAACCACAAAA	(AAAAG)_5_	54	240	Pr010317030	3	0.225
Mean						**2.75**	**0.314**
**Compound**							
sMo00152	F: GGAACAGAGGACAAGAAAGAAA R: TGTATCAAGCCTCAAGTATCTGG	(AC)6(AG)_11_	56	255	Pr009947972	3	0.209
sMo00154	F: CAAAAGGGTTGTTTGTATACGTG R: TGCATGAATATCCTCTCAAAGTTAC	(TG)_7_cgcgcgtgtgcgcgtg(TA)_8_	54	161	Pr010317031	8	0.349
sMo00161	F: ACTGTTTCGTCAAGCATTTG R: ATCAAGAGAAGGTCGTGTCAG	(TG)_8_(AG)_8_	54	163	Pr010317032	1	0
Mean						**4.0**	**0.279**

**Table 3:** Summary of observed allele numbers (*A_o_*), percentage polymorphic loci (*P*), observed and expected heterozygosity (*H*_o_ and *H*_e_) and (*F_is_*) for 14 loci across six oil palm populations.

Country	N	*A_o_*	*P (%)*	*H_o_(SD)*	*H_e_(SD)*	*F_is_*
*E. oleifera*						
Colombia	29	2.56	50.0	0.200 (0.263)	0.275 (0.325)	0.273 *
Costa Rica	34	3.00	55.6	0.160 (0.223)	0.253 (0.316)	0.368 *
Panama	34	2.89	55.6	0.193 (0.264)	0.310 (0.325)	0.377 *
Honduras	22	2.17	50.0	0.102 (0.197)	0.210 (0.260)	0.514 *
Mean		2.66	52.8	0.164 (0.238)	0.262 (0.307)	0.383
*E. guineensis*						
Deli *dura*	10	2.07	44.4	0.118 (0.171)	0.260 (0.282)	0.546 *
Nigeria	10	2.47	50.0	0.321 (0.362)	0.329 (0.305)	0.024
Mean		2.27	47.2	0.220 (0.267)	0.295 (0.294)	0.285

*P* = Percentage of polymorphic loci (0.95 criterion); *F_is_* = Inbreeding coefficient (Wright’s 1965: 1- [*Ho*/*He*]).

* significant deviation from HWE at *P* <0.01.

**Table 4:** Cross amplification of *E. oleifera g*SSR primers in various palm (Arecaceae) species.

Genus	*Elaeis*				*Cocos*			*Oenocarpus*	*Euterpe*	*Jessenia*	*Ptychosperma*	*Cyrtostachys*	*Dictyosperma*

Species	*oleifera*		*guineensis*		*Nucifera*			*multicaulis-*Spruce	*oleracea*	*bataua*	*Macarthurii*	*renda* Blume	*album*

SSR locus	Colombia	Costa Rica	Nigeria	Deli dura	*Cocos nucifera* (Yellow)	*Cocos nucifera* (Red)	*Cocos nucifera* (Green)	*Oenocarpus multicaulis-*Spruce	*Euterpe oleracea*	*Jessenia bataua*	*Ptychosperma Macarthurii*	*Cyrtostachys renda* Blume	*Dictyosperma album*
sMo00020	191	200	184	190	210	218	219	-	NA	NA	188	NA	NA
sMo00027	212	210	200	200	300	300	300	226	226	224	NA	260	200
sMo00055	200	200	188	188	195	195	195	190	190	190	190	190	190
sMo00129	222	230	204	204	178	178	178	180	NA	NA	NA	NA	NA
sMo00130	192	192	176	184	176	176	176	188	188	188	-	208	184
sMo00134	252	252–260	252	252	252–260	252–260	252–260	-	252–260	-	252–260	-	-
sMo00137	151	151	154–162	162	140	138	140	150	140	151	142	146	142
sMo00138	190–206	200	184–198	198	208	218	208	-	-	NA	186	NA	NA
sMo00140	214	214	204	204	184	184	184	204	204	-	-	-	204
sMo00141	176	176	250–260	250–260	176	176	176	NA	260	260	NA	NA	NA
sMo00154	160	160	238	232	160	160	160	160	160	160	160	160	NA

Figures given are base pair size of fragments: Missing sample; NA, not amplifiable/banding pattern not clear.

**Table 5:** Palms (family Arecaceae) analyzed using *Elaeis oleifera* gSSR markers.

Genus	Species	Full Name	Origin	No. of Palms
*Elaeis*	*Oleifera*	*Elaeis oleifera*	Colombia	29
			Costa Rica	34
			Panama	34
			Honduras	22
			Sub-total	119
*Elaeis*	*guineensis*	*Elaeis guineensis*	Nigeria	10
			Deli *dura*	10
			Sub-total	20
*Cocos*	*Nucifera*	*Cocos nucifera*	Solomon Islands	3
*Euterpe*	*Oleracea*	*Euterpe oleracea*	South America	2
*Jessenia*	*Bataua*	*Jessenia bataua*	Mart. South America	1
*Oenocarpus*	*multicaulis-*spruce	*Oenocarpus multicaulis* Spruce	North-western South America,	1
*Ptychosperma*	*macarthurii*	*Ptychosperma macarthurii*	Northeastern Australia	1
*Cyrtostachys*	*renda* Blume	*Cyrtostachys renda* Blume	Malaysia, Indonesia	1
*Dictyosperma*	*Album*	*Dictyosperma album*	Mauritius	1
			Sub-total	10
			Total	149
